# Identification and genotyping of bacteria from paired vaginal and rectal samples from pregnant women indicates similarity between vaginal and rectal microflora

**DOI:** 10.1186/1471-2334-9-167

**Published:** 2009-10-14

**Authors:** Nabil Abdullah El Aila, Inge Tency, Geert Claeys, Hans Verstraelen, Bart Saerens, Guido Lopes dos Santos Santiago, Ellen De Backer, Piet Cools, Marleen Temmerman, Rita Verhelst, Mario Vaneechoutte

**Affiliations:** 1 Laboratory Bacteriology Research, Department of Clinical Chemistry, Microbiology & Immunology, University of Ghent, Ghent, Belgium; 2 Department of Obstetrics & Gynaecology, Ghent University Hospital, University of Ghent, Ghent, Belgium

## Abstract

**Background:**

The vaginal microflora is important for maintaining vaginal health and preventing infections of the reproductive tract. The rectum has been suggested as the major source for the colonisation of the vaginal econiche.

**Methods:**

To establish whether the rectum can serve as a possible bacterial reservoir for colonisation of the vaginal econiche, we cultured vaginal and rectal specimens from pregnant women at 35-37 weeks of gestation, identified the isolates to the species level with tRNA intergenic length polymorphism analysis (tDNA-PCR) and genotyped the isolates for those subjects from which the same species was isolated simultaneously vaginally and rectally, by RAPD-analysis.

One vaginal and one rectal swab were collected from a total of each of 132 pregnant women at 35-37 weeks of gestation. Swabs were cultured on Columbia CNA agar and MRS agar. For each subject 4 colonies were selected for each of both sites, i.e. 8 colonies in total.

**Results:**

Among the 844 isolates that could be identified by tDNA-PCR, a total of 63 bacterial species were present, 9 (14%) only vaginally, 26 (41%) only rectally, and 28 (44%) in both vagina and rectum. A total of 121 (91.6%) of 132 vaginal samples and 51 (38.6%) of 132 rectal samples were positive for lactobacilli. *L. crispatus *was the most frequently isolated *Lactobacillus *species from the vagina (40% of the subjects were positive), followed by *L. jensenii *(32%), *L. gasseri *(30%) and *L. iners *(11%). *L. gasseri *was the most frequently isolated *Lactobacillus *species from the rectum (15%), followed by *L. jensenii *(12%), *L. crispatus *(11%) and *L. iners *(2%).

A total of 47 pregnant women carried the same species vaginally and rectally. This resulted in 50 vaginal/rectal pairs of the same species, for a total of eight different species. For 34 of the 50 species pairs (68%), isolates with the same genotype were present vaginally and rectally and a high level of genotypic diversity within species per subject was also established.

**Conclusion:**

It can be concluded that there is a certain degree of correspondence between the vaginal and rectal microflora, not only with regard to species composition but also with regard to strain identity between vaginal and rectal isolates.

These results support the hypothesis that the rectal microflora serves as a reservoir for colonisation of the vaginal econiche.

## Background

The composition of the human vaginal microflora is affected by several host factors, including, among others, age, menarche, sexual activity, pregnancy and the use of contraceptives or spermicides, as well as individual habits such as douching [1]. Several bacterial species are known to colonize both the gastrointestinal and the reproductive tract, and the rectum has been suggested to play an important role as a source or reservoir for organisms that colonize the vagina [2,3]. It is important to establish to which degree this is also the case for lactobacilli, the predominant group of microorganisms of the normal vaginal microflora, because these bacteria are generally known to produce endogenous microbicides such as lactic acid, which acidifies the vagina, and hydrogen peroxide (H_2_O_2_), toxic to other bacteria and viruses, including HIV [4]. Studies of vaginal lactobacilli have demonstrated that *L. crispatus, L. jensenii, L. gasseri *and *L. vaginalis *are the most commonly recovered species of H_2_O_2_-producing lactobacilli [5-9] and the absence of H_2_O_2_-producing lactobacilli in the vagina has been associated with an increased risk for bacterial vaginosis (BV) [10,11]. BV has been linked to increased shedding of HIV in the female genital tract [12], increased acquisition of HIV [10] and herpes simplex virus type 2 [10,12] and with preterm birth [13].

In order to document in more detail a possible rectal origin of the vaginal microflora, this study was set up not only to compare the bacterial species present in vagina and rectum, but in addition, to compare the genotypes of those strains belonging to species that were present simultaneously at both sampling sites of the same subject.

## Methods

### Patients

The study was approved by the research ethics committee (IRB protocol nr 2007/096) of Ghent University Hospital, Belgium. All women attending the clinic were included and participating women gave a written informed consent. Between April and December 2007, 132 paired vaginal and rectal swabs were collected from pregnant women at 35 - 37 weeks of gestation.

### Sampling procedures

All specimens were collected using nylon flocked swabs that were submerged into 1 ml of liquid Amies transport medium (eSwab, Copan Diagnostics, Brescia, It.). For rectal specimens, a swab was carefully inserted approximately 1.5 - 2 cm beyond the anal sphincter and then gently rotated to touch anal crypts.

Vaginal samples were collected by inserting a swab into the vagina. The swab was rolled round through 360 degrees against the vaginal wall at the midportion of the vault. At Ghent University Hospital, the routine screening for group B streptococci of pregnant women is always performed during the prenatal consultation at 35-37 weeks' gestation according to the CDC guidelines for the prevention of perinatal Group B streptococcal disease [14]. All study samples were collected by midwives and transported to the Laboratory for Bacteriology Research of the University of Gent within 4 hours.

### Culture and Gram staining

A total of 70 μl from the Amies liquid transport medium of each of the vaginal and rectal swabs was inoculated onto Columbia CNA agar with 5% sheep blood (Columbia CNA agar, Becton Dickinson, Erembodegem, Belgium) respectively De Mann Rogosa Sharp Agar (MRS, Oxoid, Hampshire, UK) and then incubated at 37°C in an anaerobic chamber (10% H_2_, 10% CO_2_, 80% N_2_) (BugBox, LedTechno, Heusden-Zolder, B.) for 72 h. Another 50 μl of the vaginal swab suspension was taken for smear preparation for the Gram stain.

Gram stain based grading was carried out according to modified Ison & Hay criteria [15], as described by Verhelst *et al*. [16].

Grade Ia specimens contained mainly *Lactobacillus crispatus *cell types, i.e. plump, quite homogeneous lactobacilli, grade Ib contained non-*L. crispatus *cell types, i.e. long or short, thin lactobacilli, grade Iab contained mixtures of *L. crispatus *and non-*L. crispatus *cell types, grade I-like contained irregular-shaped Gram positive rods, grade II contained a mixture of *Lactobacillus *cell types and bacterial vaginosis-associated bacteria (*Gardnerella*, *Bacteroides-Prevotella *and *Mobiluncus *cell types), whereas samples devoid of *Lactobacillus *cell types with the presence of only *Gardnerella*, *Bacteroides- Prevotella *or *Mobiluncus *cell types were classified as grade III. Finally, samples were classified as grade IV when Gram positive cocci were predominantly present and as grade 0 when no bacterial cells were present [16].

### DNA-extraction from isolates

DNA was extracted from cultured isolates by alkaline lysis as follows: One bacterial colony was suspended in 20 μl of lysis buffer (0.25% sodium dodecyl sulfate, 0.05 N NaOH) and heated at 95°C for 15 min. The cell lysate was diluted by adding 180 μl of distilled water. The cell debris was spun down by centrifugation at 16,000 *g *for 5 min. Supernatants were used for PCR or frozen at -20°C until further use.

### Identification of isolates

From all 132 women, 8 colonies per subject, i.e., one colony of each of the two most abundant colony types from both Columbia CNA and MRS agar plates and for both rectal and vaginal swabs were picked, i.e. a total of 1056 isolates. Isolates were identified by tRNA intergenic length polymorphism analysis (tDNA-PCR) as described before [8,17-19]. Briefly, the tRNA-intergenic spacer regions were amplified by PCR using consensus primers, applicable to most bacterial species, and the resulting fingerprints, obtained by separation of the amplified spacers by capillary electrophoresis on an ABI310, were compared with those of a large library of reference strains of the different species, shown in previous studies to be part of the vaginal microflora. Isolates with fingerprints that did not match fingerprints already present in the library were considered as not identifiable.

### Genotyping of isolates

Isolates of species present in both vagina and rectum of the same subject were genotyped using RAPD-analysis with RAPD Ready-to-Go beads (GE Healthcare, Buckinghamshire, UK) as described previously [20] with primer OPM1 (5' GTT GGT GGC T) at a final concentration of 2 μM, including 0.2 μM of fluorescent TET-labeled OPM1 primer. After 5 min at 94°C, 5 min at 35°C and 5 min at 72°C, reaction mixtures were cycled 30 times in a Veriti™ Thermal Cycler (Applied Biosystems, Foster City, Ca.), with the following conditions: 30 s at 94°C, 1 min at 35°C, and 1 min at 72°C, with a final extension period of 5 min at 72°C. Reaction vials were then cooled to 10°C until electrophoresis.

### Capillary electrophoresis

A volume of 11.9 μl of deionized formamide (ACE formamide, Lucron, De Pinte) was mixed with 0.6 μl of an internal size standard mixture containing 0.3 μl of the ROX-400 high-density size standard (Applied Biosystems, Foster City, Ca.) and 0.3 μl of Map marker 1000 size standard (BioVentures, Murfreesboro, Tn.). One μl of RAPD-PCR product was added. The mixtures were denatured by heating at 95°C for 3 min and placed directly on ice for at least 10 min. Capillary electrophoresis was carried out using an ABI-Prism 310 genetic analyzer (Applied Biosystems) at 60°C, at a constant voltage of 1.5 kV, and at a more or less constant current of approximately 10 mA. Capillaries with a length of 47 cm and diameter of 50 μm were filled with performance-optimized polymer 4. Electropherograms were normalized using Genescan Analysis software, version 2.1 (Applied Biosystems).

### Data analysis

tDNA-PCR and RAPD fingerprints were obtained as table files from the GeneScan Analysis software (Applied Biosystems) and analyzed with BaseHopper, an in house software program [17]. The obtained tDNA fingerprints were compared with those of a library of tDNA fingerprints obtained from reference strains, representing most vaginal species isolated in previous studies and previously identified by 16S rRNA gene sequencing [8].

Similarity between RAPD fingerprints was calculated using the Dice algorithm. Clustering analysis was done with the Neighbor module of the Phylip software http://evolution.genetics.washington.edu/phylip.html, using the Neighbour joining algorithm. Isolates of which the RAPD fingerprints were clustered together, were inspected visually to confirm similarity.

## Results

### Categorization of vaginal microflora

Samples were categorized as grade Ia for 55 subjects (41.6%), grade Ib for 37 (28.0%), grade Iab for 13 (9.8%), grade I-like for 5 (3.8%), grade II for 14 (10.6%), grade III for 6 (4.5%) and grade 0 for 2 (1.5%).

The most common genus recovered from grade Ia, Ib and Iab specimens was *Lactobacillus*. Grade Ia samples contained predominantly *L. crispatus *(75.0%) and *L. jensenii *(43.6%), whereas *L. gasseri *(40.5%) and *L. iners *(27%) were the most frequently present species in grade Ib specimens. The five grade I-like specimens were found to contain respectively *Bifidobacterium bifidum *and *Enterococcus faecalis*, *L. gasseri *and *E. faecalis*, *L. jensenii*, *L. gasseri *and *Gardnerella vaginalis *or *L. rhamnosus*.

The most characteristic cultured organisms in grade II and grade III specimens were *G. vaginalis *(28% and 33%, respectively), *Actinomyces neuii*, *Aerococcus christensenii*, *Atopobium vaginae *and *Finegoldia magna*. The lactobacilli cultured from the six grade III specimens were respectively *L. iners*, *L. gasseri*, *L. jensenii*, *L. crispatus*, *L. rhamnosus *and a combination of *L. gasseri *and *L. vaginalis*.

### Rectal and vaginal prevalence of different bacterial species

For a total of 132 women, 4 colonies each were picked from the vaginal and rectal sites, i.e. a total of 1056 colonies were picked and subjected to identification by tDNA-PCR. Of these, 844 could be identified.

A total of 103 isolates gave no amplification or tDNA-PCR patterns composed of only a few and short tRNA intergenic spacers. Most of the isolates for which no amplification or only a few fragments could be obtained, are probably corynebacteria, which yield poor tDNA-PCR fingerprints (unpublished data).

Finally, 109 isolates gave uninterpretable patterns, due to mixed cultures, as was confirmed by 16S rRNA gene sequencing for 20 of these, whereby the sequences could not be interpreted because of ambiguities, pointing to mixtures.

The frequency of vaginal and rectal colonization by lactobacilli and the other most prominent bacterial species is shown in Table 1. A total of 63 bacterial species were identified, 9 (14%) occurring only vaginally, 26 (41%) only rectally and 28 (44%) in both vagina and rectum, with 8 species that could be isolated simultaneously from rectum and vagina of 47 subjects.

**Table 1:** Vaginal and rectal prevalence of 63 bacterial species among 132 pregnant women

	Species	Only vaginal^a^	Only rectal	Vaginal + rectal	Overall
1	*Acinetobacter baumannii *group	0	8	0	8
2	*Actinomyces meyeri*	0	2	0	2
3	*Actinomyces neuii*	2	0	0	2
4	*Actinomyces radingae*	0	1	0	1
5	*Actinomyces urogenitalis*	0	1	0	1
6	*Aerococcus christensenii*	3	2	0	5
7	*Aerococcus viridans*	0	1	0	1
8	*Agrobacterium radiobacter*	0	2	0	2
9	*Alloscardovia omnicolens*	0	1	0	1
10	*Anaerococcus tetradius*	0	1	0	1
11	*Anaerococcus vaginalis*	0	4	0	4
12	*Atopobium vaginae*	2	2	0	4
13	*Bacteroides uniformis*	1	1	0	2
14	*Bifidobacterium bifidum*	2	6	1	9
15	*Bifidobacterium longum *subsp. *longum*	2	5	0	7
16	*Bifidobacterium longum *subsp. *infantis*	0	2	0	2
17	*Bifidobacterium breve*	0	1	0	1
18	*Corynebacterium accolens*	0	1	0	1
19	*Dialister *sp.	5	0	0	5
20	*Enterococcus faecalis*	5	19	2	26
21	*Enterococcus faecium*	1	3	0	4
22	*Enterococcus avium*	0	1	0	1
23	*Escherichia coli*	1	7	0	8
24	*Finegoldia magna*	2	40	0	42
25	*Fusobacterium gonidioformans*	0	3	0	3
26	*Gardnerella vaginalis*	10	2	0	12
27	*Klebsiella planticola*	0	1	0	1
28	*Lactobacillus casei*	2	1	0	3
29	*Lactobacillus fermentum*	1	0	0	1
30	*Lactobacillus coleohominis*	2	0	0	2
31	*Lactobacillus crispatus*	43	4	10	57
32	*Lactobacillus fermentum*	2	0	0	2
33	*Lactobacillus gasseri*	24	4	16	44
34	*Lactobacillus helveticus*	1	1	0	2
35	*Lactobacillus iners*	14	2	0	16
36	*Lactobacillus jensenii*	28	2	14	44
37	*Lactobacillus mucosae*	0	1	0	1
38	*Lactobacillus rhamnosus*	3	3	0	6
39	*Lactobacillus salivarius*	1	1	0	2
40	*Lactobacillus vaginalis*	13	0	0	13
41	*Lactobacillus coleohominis*	1	0	0	1
42	*Pediococcus acidilactici*	1	0	0	1
43	*Peptoniphilus indolicus*	1	25	0	26
44	*Peptostreptococcus anaerobius*	1	4	0	5
45	*Peptostreptococcus *sp.	0	8	0	8
46	*Prevotella bivia*	4	2	0	6
47	*Pseudomonas stutzeri*	0	1	0	1
48	*Staphylococcus aureus*	0	1	0	1
49	*Staphylococcus capitis*	1	4	0	5
50	*Staphylococcus epidermidis*	3	13	0	16
51	*Staphylococcus haemolyticus*	2	1	0	3
52	*Staphylococcus hominis*	1	4	0	5
53	*Staphylococcus warneri*	0	2	0	2
54	*Streptococcus agalactiae*	1	4	3	8
55	*Streptococcus anginosus*	3	44	3	50
56	*Streptococcus bovis*	0	8	0	8
57	*Streptococcus intermedius*	0	1	0	1
58	*Streptococcus mitis *group	0	7	0	7
59	*Streptococcus mutans*	1	0	0	1
60	*Streptococcus parasanguinis*	0	1	0	1
61	*Streptococcus salivarius*	3	2	1	6
62	*Varibaculum cambriense*	0	1	0	1
63	*Weissella paramesenteroides*	0	1	0	1
		193	270	50	513

a: Number of pregnant women carrying this species.

Overall, 121 of 132 pregnant women (92%) carried vaginal lactobacilli and 52 (39%) carried rectal lactobacilli. Seventy two pregnant women (54.5%) carried lactobacilli only vaginally, three only rectally (2%) and 49 (37%) in both sites, i.e. only three women out of 52 from whom lactobacilli could be isolated rectally, did not carry lactobacilli vaginally. *L. crispatus *was the most frequently identified *Lactobacillus *species isolated from the vagina (40%, i.e., 53/132 subjects positive of which 10 also carried *L. crispatus *rectally), followed by *L. jensenii *(32%), *L. gasseri *(30%), *L. iners *(11%) and *L. vaginalis *(10%).

Besides these five *Lactobacillus *species, all other species were encountered in no more than five subjects, except for *G. vaginalis *(8% of subjects positive).

*L. gasseri *was the most frequently isolated *Lactobacillus *species from the rectum (20/132 subjects positive), followed by *L. jensenii *(16) and *L. crispatus *(14). *L. iners *was isolated rectally from only 2 subjects. *L. vaginalis *was isolated only from the vagina, whereas *L. fermentum*, *L. coleohominis *and *L. fermentum *were only isolated from the rectum, at a frequency of ≤ 1%. Rectally, the most abundant species that could be cultured, were *Streptococcus anginosus *group (47/132 subjects positive), *Finegoldia magna *(40), *Peptoniphilus indolicus *(25) and *E. faecalis *(21).

Fourty six pregnant women (35%) were colonized by at least 2 different *Lactobacillus *species, with 42 of them only vaginally, two only rectally and another two both in the vagina and rectum. Taking into account vaginal and rectal colonization by more than one *Lactobacillus *species, 18 women (13.6%) were colonized by both *L. crispatus *and *L. jensenii*, of which 17 vaginally and one rectally, five (4%) by *L. jensenii *and *L. gasseri *of which 3 vaginally and 2 rectally and another 18 (13.6%) with other combinations of *Lactobacillus *species. Seven subjects (5.3%) were colonized vaginally by more than two *Lactobacillus *species. In total, of the 121 women colonized vaginally by lactobacilli, 42 (32%) were colonized by two or more *Lactobacillus *species and of the 52 women colonized by lactobacilli rectally, two (1.5%) were colonized by two *Lactobacillus *species. A total of 47 (35%) of 132 pregnant women were colonized both vaginally and rectally with the same species and 3 of these 47 women carried two species both vaginally and rectally. The species found to be simultaneously present in the same subject both rectally and vaginally were *L. crispatus *(n = 10 subjects), *L. jensenii *(14), *L. gasseri *(16), *S. anginosus *(3), *S. agalactiae *(3), *S. salivarius *(1), *E. faecalis *(2) and *Bifidobacterium *species (1). In summary, 50 vaginal/rectal pairs of the same species were observed in 47 subjects, for a total of eight different species.

### Genotyping of bacterial isolates from the vagina and the rectum

For 34 of the 50 vaginal/rectal species pairs, isolates with the same genotype were present vaginally and rectally.

Table 2 presents the genotyping results for each of the 50 vaginal/rectal species pairs. We found the same genotype for both rectal and vaginal *L. crispatus *isolates in 7/10 subjects, for *L. gasseri *in 14/16, for *L. jensenii *in 7/14, for *Bifidobacterium *species in 1/1, for *E. faecalis *in 1/2, for *S. agalactiae *in 2/3, and for *S. anginosus *in 2/3.

**Table 2:** Genotyping results for the 50 cases in which the same species could be isolated from vagina and rectum of the same subject

	Subjects, arranged per species	V1^a^	V2	V3	V4	R1	R2	R3	R4	V = R^c^
	*Lactobacillus crispatus*									
1	RVS003	**B^b^**	**A**		**C**			**A**		1
2	RVS011			**A**	**A**			**A**		2
3	RVS013		**A**	**B**				**A**		3
4	RVS020	**A**			**B**				**C**	
5	RVS023			**A**	**B**			**C**		
6	RVS028	**A**			**B**			**C**	**D**	
7	RVS043			**A**				**A**		4
8	RVS061	**A**	**A**	**A**	**A**				**A**	5
9	RVS069	**B**	**A**	**C**	**A**			**A**		6
10	RVS099	**B**		**A**					**A**	7
	*Lactobacillus jensenii*									
11	RVS015	**B**	**C**	**D**	**A**		**A**			8
12	RVS019	**B**	**A**	**C**	**A**	**D**		**E**		
13	RVS022		**A**	**A**	**B**		**A**	**A**		9
14	RVS023		**A**						**B**	
15	RVS038		**A**		**B**	**C**				
16	RVS040		**A**	**B**				**C**		
17	RVS057	**B**	**C**	**A**	**A**			**D**		
18	RVS058	**A**	**A**		**A**	**B**				
19	RVS080	**A**		**B**	**A**			**B**		10
20	RVS092				**A**				**A**	11
21	RVS093	**A**	**A**		**A**				**A**	12
22	RVS097	**C**	**A**	**B**	**A**			**A**		13
23	RVS111				**A**			**A**	**A**	14
24	RVS113			**A**		**B**				
	*Lactobacillus gasseri*									
25	RVS024			**A**		**A**		**A**	**A**	15
26	RVS025	**B**		**A**	**A**			**C**		
27	RVS031		**A**	**A**	**B**				**C**	
28	RVS035	**A**	**A**					**A**		16
29	RVS044			**A**				**A**		17
30	RVS051		**A**	**A**				**A**		18
31	RVS054			**A**					**A**	19
32	RVS060			**A**		**A**				20
33	RVS072				**A**			**A**		21
34	RVS084	**B**			**A**				**A**	22
35	RVS090			**A**	**A**			**A**	**A**	23
36	RVS091	**A**			**A**			**A**	**B**	24
37	RVS095		**B**	**A**	**A**				**A**	25
38	RVS109			**A**	**B**			**A**		26
39	RVS110	**A**	**A**	**B**	**B**			**B**	**A**	27
40	RVS136	**A**	**A**	**A**	**A**			**A**		28
	*Streptococcus anginosus*									
41	RVS003			**A**		**B**				
42	RVS058			**A**			**A**	**B**		29
43	RVS068				**A**		**A**	**A**		30
	*Streptococcus agalactiae*									
44	RVS027	**B**		**A**			**A**		**A**	31
45	RVS064	**A**	**A**	**A**	**A**			**A**		32
46	RVS076		**A**		**B**	**C**	**D**			
	*Enterococcus faecalis*									
47	RVS086	**A**	**A**	**A**	**A**	**A**	**A**	**A**	**A**	33
48	RVS122				**B**	**A**	**A**			
	*Streptococcus salivarius*									
49	RVS075				**A**				**B**	
	*Bifidobacterium bifidum*									
50	RVS033	**A**			**A**			**A**		34

a: V: vaginal isolates 1-4, R: rectal isolates 1-4.

b: Different genotypes are designated A, B, C, D or E per subject. Genotypic similarity/difference only relates to the other isolates of the same subject, i.e. genotype A of subject 1 does not indicate similarity to genotype A of subject 2.

c: Women for which the genotype of at least one vaginal isolate was identical to that of at least one rectal isolate.

**Figure 1:**
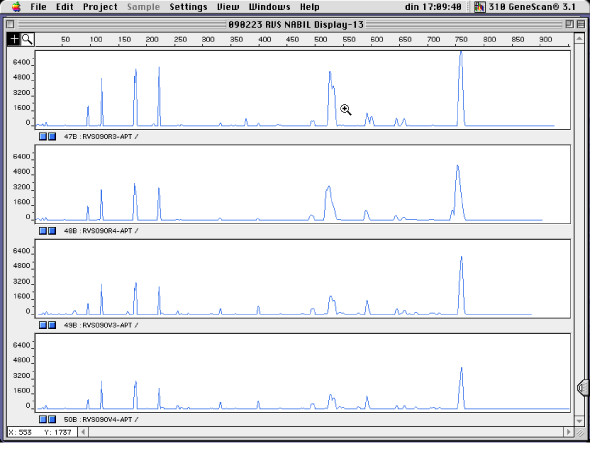
**RAPD fingerprints of vaginal and rectal *L. gasseri *isolates of subject RVS090. Vaginal isolates: V3 and V4; rectal isolates: R3 and R4**. x-axis: length of amplified DNA fragments expressed in bps. y-axis: peak height (fluorescence intensity of DNA-fragment as measured by ABI310 capillary electrophoresis).

**Figure 2:**
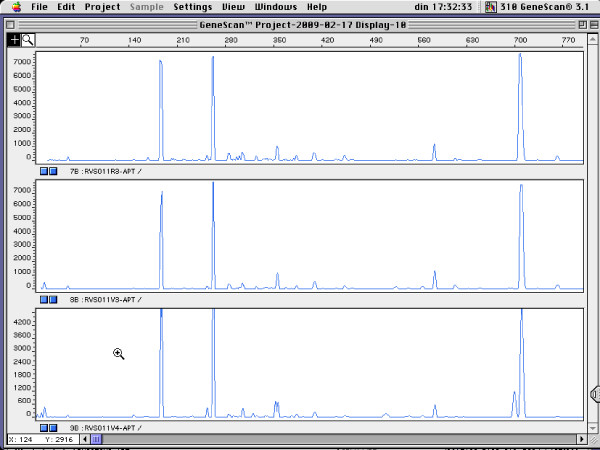
**RAPD fingerprints of vaginal and rectal *L. crispatus *isolates of subject RVS011. Vaginal isolates: V3 and V4; rectal isolate: R3**. x-axis: length of amplified DNA fragments expressed in bps. y-axis: peak height (fluorescence intensity of DNA-fragment as measured by ABI310 capillary electrophoresis).

**Figure 3:**
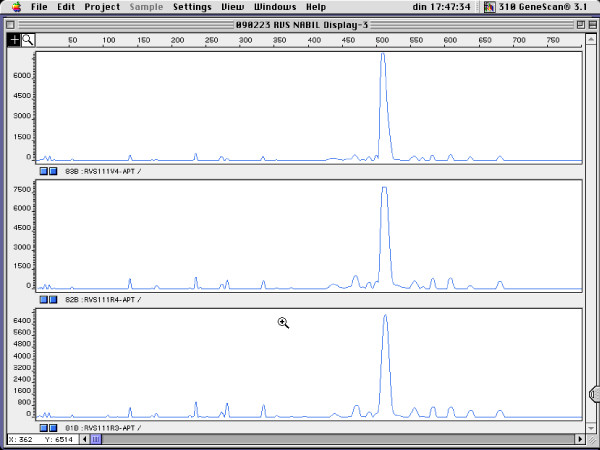
**RAPD fingerprints of vaginal and rectal *L. jensenii *isolates of subject RVS111. Vaginal isolate: V4; rectal isolates: R3 and R4**. x-axis: length of amplified DNA fragments expressed in bps. y-axis: peak height (fluorescence intensity of DNA-fragment as measured by ABI310 capillary electrophoresis).

**Figure 4:**
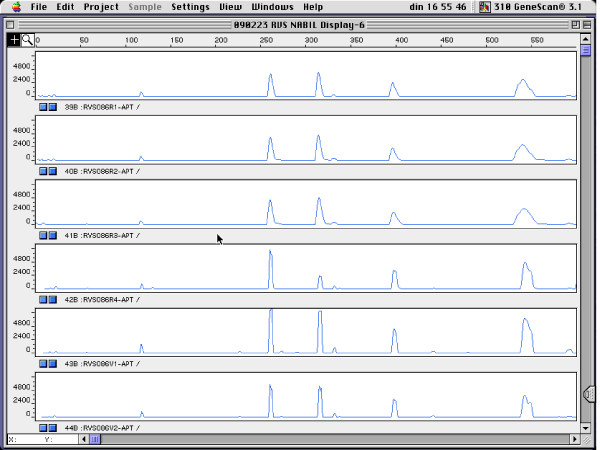
**RAPD fingerprints of vaginal and rectal *E. faecalis *isolates of subject RVS086. Vaginal isolates: V1 and V2; rectal isolates: R1 - R4**. x-axis: length of amplified DNA fragments expressed in bps. y-axis: peak height (fluorescence intensity of DNA-fragment as measured by ABI310 capillary electrophoresis).

Figure 1 shows the genotyping results for four pregnant women for which rectal and vaginal isolates belonged to a single genotype, for *L. gasseri *(Figure 1), *L. crispatus *(Figure 2), *L. jensenii *(Figure 3) and *E. faecalis *(Figure 4).

### Genotyping results: variability among multiple isolates per subject

For only 2 of the 9 vaginal samples of which more than one *L. crispatus *isolate was picked, all the isolates belonged to the same genotype. For the 7 other vaginal samples, 2 to 3 genotypes of *L. crispatus *were present. In 7 of the 10 cases for which rectal/vaginal pairs of *L. crispatus *were observed, the one or two rectal *L. crispatus *isolates had the same genotype of at least one of the one to four vaginal isolates. For only two of the 11 women for which more than one vaginal *L. jensenii *isolate was present, the genotype of all isolates was identical. For another two of these 11 women, all four isolates were genotypically different. For half (7/14) of the subjects with *L. jensenii*, the presence of identical rectal and vaginal isolates could be established.

For the other species, a similar pattern of large heterogeneity among the vaginal strains of the same species was also observed and about half of the women were found to carry at least one vaginal isolate genotypically identical to at least one rectal isolate.

## Discussion

During the last decade, the composition of the vaginal microflora has been well characterised, using culture based and culture independent methods [8,21-27]. However, the origin of lactobacilli and BV associated bacteria remains less well-understood and different opinions exist as to whether the vaginal bacteria are largely endogenous or whether there is continuous recolonizaton from the rectum. Few studies have addressed the species composition of both vagina and rectum in the same subjects [2,3,5] and only the study of Marrazzo *et al*. [3] genotyped paired rectal/vaginal isolates, from lesbian women. To our knowledge, this is the first study among pregnant women to address the relatedness of vaginal and rectal strains to the degree of clonal identity of the strains.

### Rectal and vaginal occurrence of lactobacilli

Table 3 compares the findings of this study with those of two other groups with respect to rectal and vaginal colonization by lactobacilli. The four species predominant in the vagina, as established in this study, i.e. *L. crispatus*, *L. jensenii, L. gasseri *and *L. iners *are in correspondence with previous studies [2,3,6,16,28-30].

**Table 3:** Vaginal and rectal occurrence of *Lactobacillus *species, expressed as percentage of subjects positive, according to different studies

Authors	Population studied	Species	Vagina only	Vagina all^a^	Vagina & Rectum	Rectum only	Rectum all^a^	Overall
Antonio *et al*. 1999	302 sexually active women	*L. crispatus*	32					
		*L. jensenii*	23					
		*L. gasseri*	5					
		*L. iners*	15					
		*L. vaginalis*	< 1					
Antonio *et al*. 2005	290 nonpregnant women	*L. crispatus*	17	31	14	1	15	33
		*L. jensenii*	17	23	6	4	10	27
		*L. gasseri*	3	5	2	8	10	13
		*L. iners*	15	16	1	0	1	16
		*L. vaginalis*	< 1	0	0	0	0	0
Marrazzo *et al*. 2009	237 women having sex with women	*L. crispatus*	49	93	44	5	49	98
		*L. jensenii*	8	10	2	1	3	11
		*L. gasseri*	10	16	6	6	12	21
		*L. iners*	1	1	0	0	0	1
		*L. vaginalis*	0	0	0	0	0	0
This study	132 pregnant women	*L. crispatus*	33	41	8	3	11	42
		*L. jensenii*	21	32	11	2	13	33
		*L. gasseri*	18	30	12	3	15	33
		*L. iners*	11	11	0	2	2	12
		*L. vaginalis*	10	10	0	0	0	10

a: The column entitled 'Vagina all' presents the sum of subjects with the species in the vagina only and those with the species in both vagina and rectum. The column entitled 'Rectum all' presents the sum of subjects with the species in the rectum only and those with the species in both vagina and rectum.

Both Antonio *et al*. [2] and Marrazzo *et al*. [3] reported *L. crispatus *and *L. jensenii *twice as much vaginally compared to their rectal occurrence, whereas we report four, respectively three times higher abundance of these species vaginally. Both groups also found approximately equal abundance for *L. gasseri *in rectum and vagina, whereas we could isolate twice as much *L. gasseri *from the vagina. *L. iners *was found ten times more often vaginally than rectally in this study and 20 times more vaginally than rectally in that of Antonio *et al*. [2], while this species was virtually absent from the study of Marrazzo *et al*. [3]. *L. vaginalis *was not found by Marrazzo *et al*. [3] and virtually absent in the study of Antonio *et al*. [2], whereas we found a vaginal carriage rate of approximately 10%.

The differences in *Lactobacillus *vaginal microflora between studies may be attributed to several factors. One suggestion is that the intestinal lactobacilli differ geographically [5], and the same may be true for the vaginal lactobacilli [31]. Also the populations studied differ, e.g. pregnant women in our study vs. nonpregnant women in the study of Antonio *et al*. [2] and lesbian women in the study of Marrazzo *et al*. [3]. Strong differences may exist between women, e.g. on average only 30% of the women carry *L. crispatus*, differences between Caucasian and black women have been reported [32], and differences may be present for each woman because samples can be taken during different phases of the menstrual cycle. More technically related factors concern variations in the way that samples are taken, transported and treated, the fact that culture media and incubation methods may strongly influence the outcome, e.g. incubation in an anaerobic chamber yields more *L. vaginalis *than incubation in an anaerobic jar (see below) and the use of MRS agar precludes isolation of *L. iners*, and that identification has often been based on phenotypic methods [33,34].

In addition, Kim *et al*. [35], reported that the vaginal microflora is not homogeneous throughout the vaginal tract but differs significantly within an individual with regard to anatomical site and sampling method used.

Apparently, when based on culture, the vaginal carriage rate for *L. crispatus *ranges between 20 and 40% (Antonio et al. [6], Kiss et al. [28], this study), although Marrazzo *et al*. [3] report a carriage rate of approximately 65%. The high percentage reported by the latter group could be related to the study of a different population (lesbian women), to the use of culture methods better suited for *L. crispatus *and corresponds better with results obtained by non culture based methods, as reported previously [16,22,26,27,32,36-38].

Interestingly, in a previous study of our group [8], we isolated almost no *L. vaginalis*, using anaerobic jars and GasPak (Becton Dickinson), yielding an atmosphere of 15% CO_2_, 80% N_2_, and less than 1% O_2_. Since we started using an anaerobic chamber, with an atmosphere of 10% H_2_, 10% CO_2_, and 80% N_2_, the number of *L. vaginalis *isolations has increased significantly and this species is now among the five most abundant vaginal species. Possibly, the virtual absence of *L. vaginalis *in the studies of Antonio *et al*. [2] and Marrazzo *et al*. [3] might be explained by the use of anaerobic culture in jars.

Comparing the reported culture results for *L. iners *remains also problematic, because this species does not grow on MRS agar, specifically designed to culture lactobacilli, and the small colonies it forms on most media may be more easily overlooked.

The rectal occurrence of lactobacilli in culture-based studies may be underreported. Because of lactobacilli are the predominant species vaginally, they can be easily overgrown by the predominant rectal bacteria as they represent about 0,01% of the overall cultivable bacterial intestinal population [39,40]. However, high occurrence of intestinal lactobacilli has been reported [41,42]. The difference with our study might be explained by the fact that these studies used fecal samples, whereas we started from rectal swabbing.

The virtual absence of *L. iners *from the rectum in this culture-based study is in correspondence with the findings of the other culture-based studies [2,3]. However, preliminary data, obtained by analyzing the same samples using *L. iners *specific realtime PCR, indicate that for most women for which this species could be isolated by culture from the vagina, also the rectal sample is *L. iners *PCR positive (data not reported).

In this population of pregnant women, we isolated lactobacilli more frequently from the vagina (121 subjects, 91.6%) than from the rectum (52 subjects, 39.3%), which is in correspondence with the findings of Antonio *et al*. [2], who reported vaginal recovery of lactobacilli in 74% and rectal recovery in 51% of a total of 531 nonpregnant females.

Although for many women from which lactobacilli could be isolated from the vagina, no lactobacilli were isolated rectally, most of the women that carried lactobacilli rectally, also had vaginal lactobacilli, i.e. few women carried lactobacilli only rectally.

### Number of *Lactobacillus *species per individual

In our study, a total of 46 (35%) of 132 pregnant women carried two or more *Lactobacillus *species vaginally and/or rectally. Marrazzo *et al*. [3] reported 72% of 237 participants to be colonized overall by lactobacilli and 24% to be overall colonized with more than one *Lactobacillus *species. Antonio *et al*. [6] reported that 8% carried more than one *Lactobacillus *species in the vagina.

A total of 18 women in this study were colonized by both *L. crispatus *and *L. jensenii *(17 vaginally and one rectally). Vaginal colonization of women with *L. crispatus *and *L. jensenii *has been suggested to be advantageous in the maintenance of a normal microflora and the prevention of sexually transmitted diseases [2,6].

### Genotyping results: clonal identity between vaginal and rectal isolates

RAPD was used in this study to detect genotypic similarity of vaginal and rectal strains of the same bacterial species. We could show that for 34 of the 50 pairs (68%) for which several isolates of the same species were present both in vagina and rectum, genotypic identity could be observed between at least one of the vaginal and at least one of the rectal isolates. In another study, on the same population of women, we compared the genotypes of rectal and vaginal *Streptococcus agalactiae *(group B streptococci: GBS) isolates and found clonal identity between isolates from both sites in 18 of the 19 subjects [43], confirming that also for GBS the rectally occurring strains are frequently identical to their vaginal counterparts. Because of the close proximity of the rectum to the vagina, the isolation of H_2_O_2_-producing vaginal *Lactobacillus *species from the rectum suggests that it may play a role as a reservoir for these microorganisms [2].

Vaginal colonization by *Lactobacillus *species was found to be transient in many females [7], and the rectum may be a source for vaginal recolonisation by lactobacilli after a disturbance of the ecology that follows douching, menses or sexual intercourse. Studies conducted between 1960 and 1980 indicated that while most *Lactobacillus *strains found in the human intestinal tract are allochthonous, *L. acidophilus, L. fermentum *(now *L. reuteri*) and *L. salivarius *can be isolated from individuals over longer periods [44-48]. Since on the basis of current taxonomy *L. crispatus *[49], *L. gasseri *[50] and *L. iners *[51] belong to the *L. acidophilus *complex the rectum may be a source for vaginal recolonization by these *Lactobacillus *species.

### Genotyping results: variability among multiple isolates per subject

We found a surprising high genotypic heterogeneity within species. For the 50 species for which isolates were available from both vagina and rectum of a total of 47 pregnant women, on average of 2.2 vaginal respectively 1.3 rectal isolates were genotyped and on average 1.6 vaginal genotypes and 1.1 rectal genotypes were found for these species. It can be expected that more species pairs, more intraspecies genotypic diversity and more identical genotypes in rectum and vagina will be found, when more isolates would be picked. Although we did not sample the same subjects at different time intervals, this finding suggests the occurrence of changes in the composition of the vaginal microflora, whereby different strains of a limited number of species may replace each other, and are may be exchanged between vagina and rectum.

The high transmissibility of strains was also established in the study of Marrazzo *et al*. [3] by the observation that many sexual partners carried genotypically identical strains. This hypothesis may be confirmed by long term follow up of individual women, e.g. during subsequent menstrual cycles, and by picking more isolates per subject and per site at multiple time points.

## Conclusion

Although it has been claimed that the vaginal microflora originates from the rectal microflora, this is to our knowledge only the second study, besides the recent study of Marrazzo *et al*. [3] to address this in detail at the strain level. Several of the total of 63 species identified were found only vaginally (9, i.e. 14.3%) or only rectally (26, i.e. 41.3%), but 29 species (44.4%) were isolated from both sites, indicating that many species can colonize the vagina from the rectum or migrate to the rectum from the vagina. For the 8 species for which isolates were present simultaneously in the same subject in vagina and rectum, we found considerable genotypic diversity within the species (i.e. on average 1.36 genotypes for on average 1.79 isolates per subject), both in rectum and vagina, as well as identical genotypes present simultaneously in rectum and vagina for 70% of the 50 species pairs studied. All these data indicate a strong correlation between vaginal and rectal microflora, not only at the species level but also at the strain level.

It is possible that the rectal colonization by lactobacilli may function as a reservoir for the maintenance of a normal vaginal flora and that this may be associated with a decreased incidence of BV-associated adverse effects, as has been suggested [3].

## Competing interests

The authors declare that they have no competing interests.

## Authors' contributions

NE, RV, GC and MV participated in the development of the study design, the analysis of the study samples, the collection, analysis and interpretation of the data, and in the writing of the report. IT, HV and MT participated in the development of the study design, the collection of the study samples, the collection, analysis and interpretation of the data, and in the writing of the report. BS, PC, GLSS and EDB participated in the analysis of the study samples and interpretation of the data. All authors read and approved the final manuscript.

## Pre-publication history

The pre-publication history for this paper can be accessed here:

http://www.biomedcentral.com/1471-2334/9/167/prepub
